# A crisis in comparative psychology: where have all the undergraduates gone?

**DOI:** 10.3389/fpsyg.2015.01500

**Published:** 2015-10-02

**Authors:** Charles I. Abramson

**Affiliations:** Department of Psychology, Oklahoma State UniversityStillwater, OK, USA

**Keywords:** comparative psychology, recruitment, undergraduates, graduate programs, education

## Introduction

Comparative psychology can generally be defined as the branch of psychology that studies the similarities and differences in the behavior of organisms. Formal definitions found in textbooks and encyclopedias disagree whether comparative psychologists restrict their work to the study of animals or include the study of human behavior. This paper offers an opinion on the major problem facing comparative psychology today—where we will find the next generation of comparative psychology students. Something must be done before we lose access to an entire generation of psychology undergraduates. It is not enough to say that comparative psychology is being “absorbed” in other academic units such as “integrative biology,” “integrative study of animal behavior,” “evolutionary psychology,” “comparative cognition,” or “animal cognition.” Indeed, many of these topics are not primarily studied in psychology departments, wherein comparative psychology students have traditionally received joint training in the principles of behavior and comparative analysis, as applied to both humans and other animals.

Consider, for example, the difference between comparative psychology and comparative cognition. Comparative cognition includes features of comparative psychology, but they are not identical. Comparative cognition represents a very specific theoretical position whose validity is based on certain suppositions such as the belief (by definition) that the internal cognitive process of non-human animals can be studied scientifically. It is unclear whether the study of behavior in comparative perspective, without reference to cognition, would fit within this endeavor at all. Conversely, comparative psychology represents a broader scientific field encompassing any number of theoretical perspectives that can be employed to make inter-species phenotypic comparisons (e.g., behaviorist, cognitive, physiological, and evolutionary). Therefore, to reduce comparative psychology to comparative cognition would foreclose upon a large portion of possible theory space. Moreover, given that undergraduates are heavily influenced in their choice of discipline by the existence of curricular specializations, the increasing paucity of opportunities to undertake a program of comparative psychology is likely driving students—and psychology majors in particular—away from engagement with this important field. Comparative psychology as a separate and distinct discipline was a vital and important branch of psychology and can be so once again if we act soon.

Comparative psychology has much to offer undergraduate students with regard to the development of critical thinking skills, personal exploration, cultivating a comprehensive view of the world around them, and the ability to apply their skills in both academic and applied fields (Moran, 1987; White, 2007). Many contemporary problems such as racism and gender bias would be better addressed by using the methods of comparative psychology (Abramson and Lack, 2014). Comparative psychologists are specifically trained to make valid comparisons and to expose those that are invalid. Training in comparative psychology is a fine example of the importance of a liberal arts education.

Many authors have commented on the problems associated with comparative psychology. These include the use of a restricted number of species (Beach, 1950; Bitterman, 1965), lack of an appreciation of evolutionary theory (Lockard, 1968; Hodos and Campbell, 1969; Kalat, 1983), decline in the number of animal facilities available for comparative research (Gallup and Eddy, 1990), scientists who begin their career as comparative psychologists only to change disciplines (Dewsbury, 1990), the expense and resources needed to fund a comparative program (Dewsbury, 1992; Varnon and Abramson, 2013), few articles containing more than one species (Lester, 1973), whether animals are needed for psychological research (Bowd, 1980; Robinson, 1990), and a lack of jobs (Dewsbury, 1990). All of these issues have contributed to the crises we now face.

## Problems recruiting students:

### Few colleges and universities offer courses in comparative psychology

In 2014, we surveyed the undergraduate course catalogs of the 650 academic institutions that *Forbes Magazine* considers the best. Of the course catalogs searched, only 82 (12.6%) offer an undergraduate course! This figure is probably overestimated because we could not determine if the course is actually taught. Certainly one can reasonably argue that the information obtained in a comparative course is included in other course offerings such as evolutionary psychology, animal behavior, and learning processes. This may be so, but as I mentioned in the introductory comments comparative psychology is unique.

### Only one currently available comparative psychology text

The last text titled “comparative psychology” was written by Papini (2002/2008) Even if Papini can still be found in enough quantities to meet class demands, for the sake of diversity of ideas and approach, alternatives texts should be available.

### Few graduate programs specifically called comparative psychology

The website “Psychology Career Center” lists only two programs, one at the University of Mary Hardin-Baylor (Belton, TX), and the other Western Washington University (Bellingham, WA). It is interesting to note that there is not a graduate program in comparative psychology at the very institutions where the last two comparative texts were written (Texas Christian University and Wichita State University).

### Comparative psychology as portrayed in introductory psychology texts

Comparative psychology is seldom mentioned in introductory psychology textbooks. This is extremely unfortunate because introductory texts provide the initial source materials for students. They also serve an important function for students because they help identify possible careers (Coleman et al., 2000; Abramson and Place, 2005).

We examined 13 contemporary introductory textbooks (Huffman, 2007; Gray, 2011; Lilienfeld et al., 2011; Krause and Corts, 2012; Wade and Tavris, 2012; Zimbardo et al., 2012; Hockenbury and Hockenbury, 2013; King, 2014; Okami, 2014; Schacter et al., 2014; Wood et al., 2014; Ciccarelli and White, 2015; Weiten et al., 2015) to find whether comparative psychology is mentioned. Only four mention comparative psychology! Even here, the descriptions are very brief and highlighted the research of a particular comparative psychologist, or comparative psychology is discussed within the context of ethics in research and/or included in a brief comment on the various areas of psychology (Huffman, 2007; Hockenbury and Hockenbury, 2013; Wood et al., 2014; Ciccarelli and White, 2015). Nothing in these descriptions encourages the reader to learn more about comparative psychology.

## Some suggestions on recruiting students:

### Recognize that comparative psychology is connected to human behavior

Our most egregious error is not making it fundamentally clear to students that comparative psychology is concerned with human behavior. Over the years, I have asked students at the beginning of the semester what they think comparative psychology is. The vast majority believe that the course will focus on how the comparative method is applied, for example, to an analysis of culture and social practices and how these human behaviors relate to those found in animals. Others have comment on the philosophical implications. The comparative analysis of human and animal behavior is one of the major philosophical controversies in the intellectual tradition of the West (Muckler, 1963).

In addition to philosophical considerations, students must be taught that the research of many “animal psychologists” clearly makes explicit that their work is designed to be integrated with human behavior. Examples include the work of Washburn (1908); Watson (1914); Keller (1937); Warden (1928), and those of the neo-behaviorists such as Abram Amsel, Clark Hull, Neal Miller, O. H. Mower, Kenneth Spence, and Edward Tolman, (Abramson, 2013). A similar connection can be found in the comparative texts of Warden et al. (1935); Stone (1951); Denny and Ratner (1970); Razran (1971); Lester (1973) and the out of print *Comparative Psychology: A Handbook* edited by Greenberg and Haraway (1998).

### Comparative psychology develops broad skills

Students must be taught that comparative psychology meets the need of employers that are looking for trained individuals with broad-based problem solving ability. Undergraduate students in comparative psychology learn about analogies, homologies, subject variables, environmental variables, observation skills, etc…They are confronted with fascinating challenges in experimental design, apparatus construction, and data interpretation. An undergraduate student with a degree emphasizing comparative psychology will be in high demand in the business world.

### Encourage students interested in comparative psychology to tailor their own study program

A student interested in comparative psychology can tailor their own plan of study in collaboration with their mentor/advisor. Such a plan can include courses such as comparative, cross-cultural, developmental, experimental design, history, learning, psychology of aging, and quantitative methods. In addition, they can take courses in animal behavior, behavioral genetics, economics, evolution, introductory biology, and neuroscience. Students should also be urged to take courses with the word “comparative” in the title such as comparative anatomy and physiology, comparative anthropology, comparative literature, comparative philosophy, comparative politics, and comparative religion. A course in apparatus design and fundamental electronic/computer programing skills would also be important to round out their plan of study.

### Development of on-line courses in comparative psychology

The number of on-line courses offered by psychology departments is increasing. There is little doubt that on-line courses should be part of the future of comparative psychology course offerings. Many universities offer development fees for the creation of on-line courses and have the suitable production facilities.

### Faculty should take advantage of every opportunity to highlight the value of comparative psychology

Dr. Kit Nast seeks out faculty and graduate students to highlight possible careers in psychology; comparative psychology is among the videos (www.drkit.org/psychology). Our laboratory regularly presents “comparative psychology” shows throughout the state of Oklahoma to encourage high school students to become interested in comparative psychology. We also participate in the annual Women in Science program, funded by EPSCoR. Another suggestion is for faculty to present at “psychology club” events (Satterfield and Abramson, 1998). I would also recommend those interested in finding graduate students to submit a grant to the National Science Foundation—Research Experience for Undergraduates Program with a comparative focus (Page et al., 2004).

### The need to develop teaching exercises for comparative psychology

I believe that the lack of student exercises contributes to the decline of comparative psychology. Over the years, I have developed classroom exercises using both invertebrates and vertebrates Abramson, 1986, 1990; Abramson et al., 2011a,b. An activity that can be performed outside of the class is what we call “project petscope” (Abramson et al., 1999). In project petscope, the local pet store becomes a comparative behavior research center. Another activity is called “correspondence in the classroom” where students are encouraged to write letters to comparative psychologists (Abramson and Hershey, 1999). Another interesting exercise is to turn comparative psychologists into official United States Postage Stamps. The stamps can include QR codes that, when scanned, lead the user to websites (Abramson and Long, 2012). We have also adapted the Parallax Propeller microcontroller (Parallax Inc.; Rocklin, California) for comparative psychology. A comparative laboratory can literally be placed in the palm of your hand and carried from office to classroom. We have developed a full range of teaching related programs that are freely available (Varnon and Abramson, 2013).

## Discussion

Dr. Donald Dewsbury writes about the history of comparative psychology and the issues which confront us. In a chapter devoted to the retrospect and prospect of comparative psychology (Dewsbury, 1990) he states: “There are no intellectual reasons that progress in comparative psychology should not continue. Comparative psychologists have made effective use of available resources in advancing their science and have produced research results and principles demonstrating the utility of their approach” (p. 447). In the next paragraph he goes on to identify what he considers to be one of the major threats to comparative psychology “Perhaps the major internal threat to the continued advancement of comparative psychology is growing fractionalization.” (pp. 447–448). While these threats continue to be faced by comparative psychologists in the twenty-first century, there is one threat that has not to my knowledge been recognized or addressed before now—where is the next generation of comparative psychologists going to come from?

We must develop a cadre of undergraduates that can fill our graduate programs. What graduate programs? Where are they? As I reported earlier, there are only a handful of graduate programs in comparative psychology and some of these are not PhD programs. While I am cognizant of the many difficulties associated with creating a comparative psychology graduate program, at Oklahoma State University, we have addressed this problem by going to a track system with comparative-neurobiology as one of the tracks. While we actually have three comparative psychologists among our faculty, this is not enough to have a viable comparative program. As a result, we have recruited “affiliated faculty” from all over the university (and outside it) to serve as advisors and to offer courses. Our students are able to take courses from such departments as animal science, engineering, human development, philosophy, sociology, veterinary medicine, and zoology. The track system in conjunction with the use of affiliated faculty may be a model suitable for many other universities. If any faculty member needs assistance in implementing any of the ideas presented in this paper, I will gladly assist.

### Conflict of interest statement

The author declares that the research was conducted in the absence of any commercial or financial relationships that could be construed as a potential conflict of interest.
